# The Beneficial Effect of a TPMS-Based Fillet Shape on the Mechanical Strength of Metal Cubic Lattice Structures

**DOI:** 10.3390/ma17071553

**Published:** 2024-03-28

**Authors:** Christian Iandiorio, Gianmarco Mattei, Emanuele Marotta, Girolamo Costanza, Maria Elisa Tata, Pietro Salvini

**Affiliations:** 1 Department of Enterprise Engineering, University of Rome “Tor Vergata”, Via del Politecnico 1, 00133 Rome, Italy; emanuele.marotta@uniroma2.it (E.M.); salvini@uniroma2.it (P.S.); 2 Department of Industrial Engineering, University of Rome “Tor Vergata”, Via del Politecnico 1, 00133 Rome, Italy; gianmarco.mattei@alumni.uniroma2.eu (G.M.); costanza@ing.uniroma2.it (G.C.); elisa.tata@uniroma2.it (M.E.T.)

**Keywords:** metal cubic lattice structures, triply periodic minimal surfaces (TPMS), lost-PLA casting, mechanical strength of lattice structures, finite element analysis, experimental tests

## Abstract

The goal of this paper is to improve the mechanical strength-to-weight ratios of metal cubic lattice structures using unit cells with fillet shapes inspired by triply periodic minimal surfaces (TPMS). The lattice structures here presented were fabricated from AA6082 aluminum alloy using lost-PLA processing. Static and dynamic flat and wedge compression tests were conducted on samples with varying fillet shapes and fill factors. Finite element method simulations followed the static tests to compare numerical predictions with experimental outcomes, revealing a good agreement. The TPSM-type fillet shape induces a triaxial stress state that significantly improves the mechanical strength-to-weight ratio compared to fillet radius-free lattices, which was also confirmed by analytical considerations. Dynamic tests exhibited high resistance to flat impacts, while wedge impacts, involving a high concentrated-load, brought out an increased sensitivity to strain rates with a short plastic deformation followed by abrupt fragmentation, indicating a shift towards brittle behavior.

## 1. Introduction

In the last several years, researchers have focused their attention on the production of a wide range of metallic and polymeric cellular materials with the aim of developing lightweight structures with adequate stiffness and strength [1,2,3,4,5]. These cellular materials can be described as porous, consisting of a network of interconnecting elements. Thanks to their lightness, high specific strength, high toughness and excellent energy absorption, cellular materials are frequently employed as core materials for sandwich structures. In these configurations, the support skins provide additional strength in response to bending and elongation, while the cores are primarily responsible for carrying compressive or impulsive loads [6,7] and keeping the skins away from each other.

The interest in lightweight materials and structures is constantly growing due to the increasing demand for sustainable products, which require reducing the amounts of materials used, as well as energy consumption and gas emissions, while maintaining high mechanical performances [8]. This approach finds application in several industrial sectors, including the aerospace, automotive [9,10,11] and biomedical industries [12]. In the field of biomedicine, for example, bones and dental grafts are being developed with lightweight, high-performance materials customized for the specific needs of patients [13,14]. In the aerospace field, cellular structures are the basis of numerous components thanks to their ability to efficiently react to compressive and impulsive loads and their extreme lightness, which are fundamental requirements for this sector. Cellular structures are also increasingly being adopted in sports competitions [15,16].

To achieve the aforementioned goals, several experimental, analytical and numerical methods have been employed to predict the topological configurations or spatial distributions of the materials necessary to achieve specific mechanical performances [1,13,17]. However, sophisticated configurations and/or complex spatial distributions of materials are often limited by traditional manufacturing technologies. Due to the evolution of production methods, in particular, additive manufacturing (AM), it is nowadays possible to produce lattice structures by adding materials layer by layer, allowing the creation of 3D architectural configurations and reducing waste with high geometric precision [18]. AM offers greater design freedom to develop new structures and materials with improved mechanical properties. Another important feature is the ability of these materials to absorb energy under high strain rates [19]. This characteristic is particularly important in industrial applications, such as the automotive and railway industries, where materials and structures must be able to absorb a considerable amount of energy through plastic deformation and/or fracture to reduce the risk of injury to people and damage to goods during impacts or collisions [20,21,22,23]. Additive manufacturing has pioneered the design and production of various new materials and structures, such as shell lattices, bio-inspired designs and shape-optimization-based structures, which would be difficult to achieve with traditional manufacturing techniques, enabling a notable improvement of their mechanical characteristics [24]. However, some issues related to the AM process are also being addressed, such as product uncertainties and defects and their effects on mechanical properties [25,26,27]. To fully understand the mechanical characteristics of these AM-based materials and structures, computational modeling of the AM process and the deformation behavior of the materials has become an indispensable tool.

Many researchers [28,29,30,31,32,33,34] have focused on improving mechanical strength-to-weight ratios using topology modification and/or optimization of the geometries in lattice structures, e.g., cubic, honeycomb, octet, Kelvin cell, etc. More recently [35,36,37], there has been a growing interest in studying how the fillet radius influences and enhances the mechanical strength and fatigue life of lattice structures. This paper addresses this issue, exploring the possibility of improving the mechanical strength-to-weight ratios of metallic cubic lattice structures using a suitable fillet shape inspired by triply periodic minimal surfaces (TPMS) [38]. The beneficial effects of this type of fillet shape are discussed in relation to static and dynamic experimental tests conducted on cubic lattice structures produced with aluminum 6082 alloy by means of the lost-PLA technique. Furthermore, Finite Element Analysis (FEA) was employed to understand the reliability of a numerical model in relation to the phenomena and structures under investigation.

## 2. Design of the TPMS Fillet Shape

A lattice structure involves the repetition and merging of a unit cell (or fundamental unit), which is the principal element that defines and characterizes the entire lattice structure, according to an ordered scheme. In the case analyzed in this paper, the lattice design entails the definition of the fillet shape, which is the core of the unit cells, and then proceeds with the creation of the lattice structure.

A promising way to define the (external) fillet shape is to take inspiration from TPMS [38,39,40,41,42,43,44], which are minimal surfaces (so they have minimal material requirements), i.e., they are characterized by zero mean curvature at every point. TPMS-based lattices demonstrate mechanical properties that potentially surpass those of other lattice structures, even if they are more complex to realize, thanks to the stiffening due to the in-plane stretching induced by bending [45,46]. Clearly, the cubic lattices addressed here involve a number of fillet shapes in a volume. The basic idea is that if the external boundary is a TPMS, this leads to a suitable triaxial distribution of internal stresses within the fillet, potentially resulting in improved mechanical performances both in the elastic range and beyond it.

A way to obtain a TPMS surface employs the Fourier series as follows [47,48,49]:

(1) $$\Psi \left(r\right)=\underset{i}{\sum }F\left(k_{i}\right)\cos\left[2\pi k_{i}\cdot r-\alpha \left(k_{i}\right)\right]=0$$

where $r=\left(x,y,z\right)$ is the radius vector; $k_{i}$ is the lattice vector (i.e., a wave vector); and $\alpha \left(k_{i}\right)$ and $F\left(k_{i}\right)$ are the phase shift and structure amplitude, respectively, both associated with a given $k_{i}$.

The minimal fillet shape considered in this paper has the following form [14]:

(2) $$F\left(x,y,z\right)=\cos\left(2\pi x\right)+\cos\left(2\pi y\right)+\cos\left(2\pi z\right)+a\;\left[\cos\left(2\pi x\right)\cos\left(2\pi y\right)+\right.\;\left.+\cos\left(2\pi y\right)\cos\left(2\pi z\right)+\cos\left(2\pi z\right)\cos\left(2\pi x\right)\right]+b\leq 0$$

The above Equation(1) is the mathematical representation of a periodic volume in implicit form, based on the two parameters a and b, resulting in a shape that is similar to but different from the Schwarz primitive structure [48,49,50].

**Figure 1:**
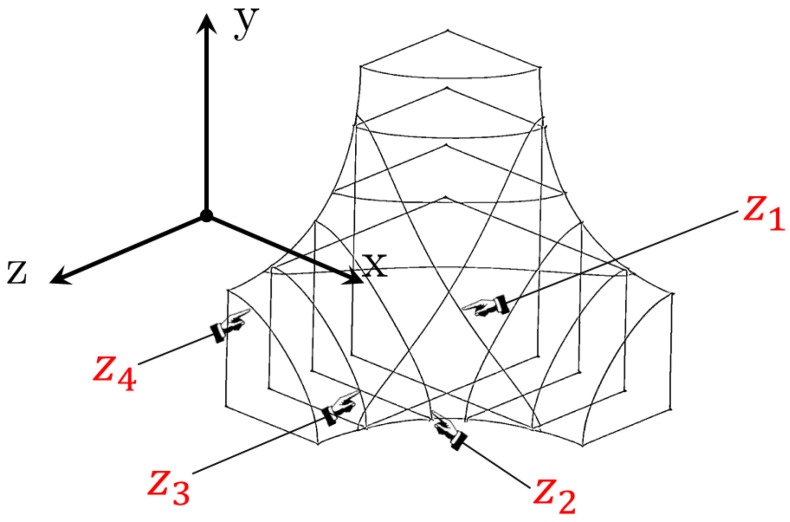
Splines defining the eighth of the cell.

To produce a lattice structure (Section 3) and perform an FE analysis (Section 4), it is essential to generate a CAD model of the unit cell, which includes the fillet shape, to form the entire lattice structure through repetitions of the unit cell. However, many CAD software packages work with the boundary representation (B-rep) and therefore are not able to directly work with the implicit geometry of Equation (1). For this reason, it is helpful to describe how to generate a CAD model of the fillet shape by dissecting Equation (2) to extract some main curves that serve as a basis to create a volume geometry using B-rep-based CAD software. Using the symmetry of the geometry, it is very useful to work only on an eighth of a unit cell and then generate the complete cell by mirroring. An eighth of a unit cell rests on the origin of the reference system, x, y, z; therefore, it is possible to identify four planes for each direction having as normal vectors the directions x, y and z. The four planes having normal vectors collinear to the z-axis are chosen at specific $z^{*}=\left\{z_{1},z_{2},z_{3},z_{4}\right\}$ coordinates (Figure 1) such that the sections of the external fillet surface, obtained by setting Equation (2) to equal zero, generate closed curves:

(3) $$\left\{\begin{array}{c} 0<z_{1}<z_{2}<\frac{arccos\left(a-b\right)}{2\pi }\\ \frac{1}{2}+\frac{arccos\left(b-a\right)}{2\pi }<z_{3}<z_{4}<1 \end{array}\right.$$

Equation (3) give the relationship between parameters a and b, which are assumed to be positive in what follows:

(4) a−1<b<1−a

The four curves of the external boundary at the z* locations (Figure 1) can be expressed by means of Equation (2) as follows:

(5) $$y\left(x,z^{*}\right)=\frac{1}{2\pi }\arccos\left\{\frac{b+\cos\left(2\pi z^{*}\right)+\cos\left(2\pi x\right)\left[1+a\cos\left(2\pi z^{*}\right)\right]}{1+a\left[\cos\left(2\pi x\right)+\cos\left(2\pi z^{*}\right)\right]}\right\}+\frac{1}{2}$$

where $x\in \left[x_{\min},x_{\max}\right]$, where

(6) $$\left\{\begin{array}{l} x_{\min}\left(z^{*}\right)=\frac{1}{2\pi }\arccos\left\{\frac{1+\left(a-1\right)\cos\left(2\pi z^{*}\right)-b}{1-a\left[1-\cos\left(2\pi z^{*}\right)\right]}\right\}\\ x_{\max}\left(z^{*}\right)=1-x_{min}\left(z^{*}\right) \end{array}\right.$$

Equations (3), (5) and (6) can be used to define the four curves along the four planes having the z-normal vector. Due to the symmetry of Equation (2), the same procedure can be used by permuting the variables x,y and z to obtain the other eight curves along the planes with the x and y normal vectors. In this manner, an eighth of a unit cell can be created in B-rep CAD software by means of B-splines. Figure 1 shows the twelve B-splines as well as the other edge lines necessary to close the unit cell geometry and to create the full volume.

**Figure 2:**
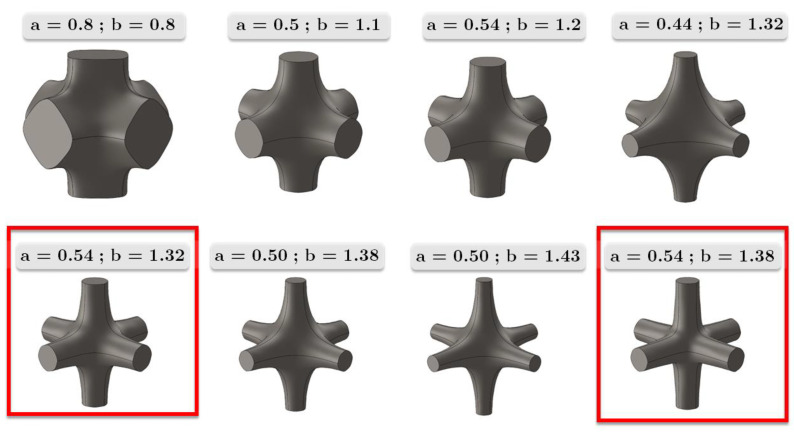
Some fillet shapes and unit cell geometries upon variation of parameters a and b. Red squares identify the unit cells studied in this paper.

Figure 2 shows the unit cell geometries computed for some parameters, a and b; their role is fundamental inasmuch as they modify the mechanical strength-to-weight ratio of the lattice structure.

**Figure 3:**
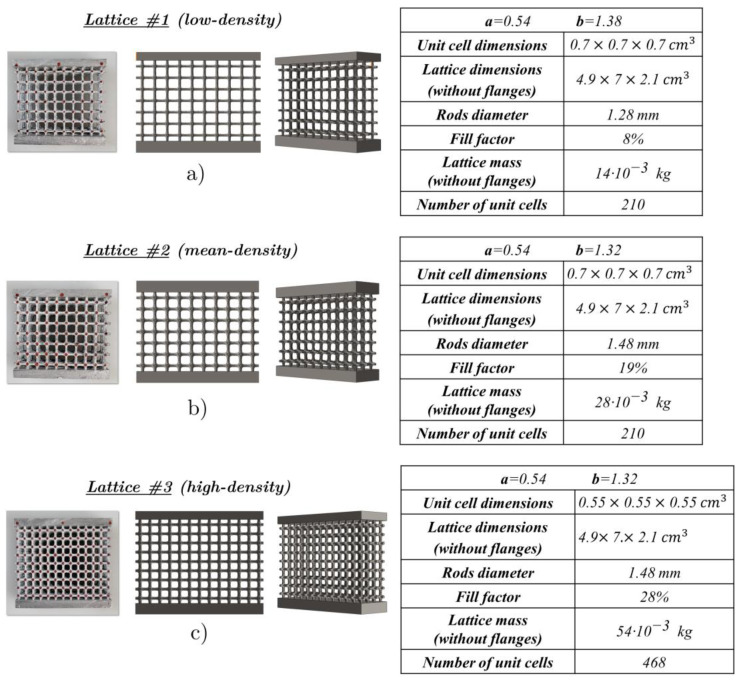
Samples of the three lattice structures examined and their CAD models: (**a**) low-density, (**b**) medium-density and (**c**) high-density structures.

In this work, three types of lattices are examined, and the geometrical characteristics (rod diameters; measurements of height, width and length; the fill factors computed as the ratio between the full volume and the total volume; the numbers of unit cells) are reported in Figure 3. All of the lattices have the same overall sizes. The first lattice (Figure 3a), with the parameters a=0.54 and  b=1.38, is characterized by a low density, while the second one (Figure 3b) and the third one (Figure 3c), with the parameters a=0.54 and  b=1.32, have medium and high densities, respectively.

The second and third lattices differ in scale; in fact, the unit cell dimension of the third lattice was scaled with respect to the second, keeping the same rod diameters, as specified in the tables of Figure 3. This allowed the dimensions of the two specimens to be identical, but the lattice in Figure 3c has a higher number of cells, thereby contributing to a significant increase in relative density and a reduction in unit cell size.

To perform flat and wedge compression tests, two supporting skins were created at the top and bottom of the lattice structures during the casting process. This resulted in a sandwich-type structure, where the supports formed planes for load applications.

## 3. Lattice Manufacturing by Lost-PLA Production and Mechanical Properties of AA 6082 Alloy after Casting

**Figure 4:**
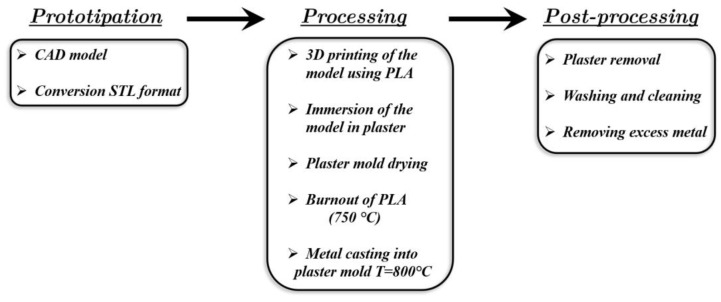
Sketch of the lost-PLA processing for the manufacture of the Al lattice structures.

The lost-PLA process is a manufacturing method inspired by an ancient process called “lost wax”, in which PLA (polylactic acid) replaces the wax. The subsequent manufacturing steps, adopted to build up the aluminum (Al) alloy samples, are summarized in the scheme of Figure 4. The starting point is a CAD model, which is converted into STL format in order for it to be processed and printed by a 3D printer. Successively, the PLA sample is immersed in liquid plaster and, after drying, the PLA is removed by burning the sample in an oven at a temperature of up to 750 °C so that the negative shape of the model remains. Clearly, the burn-in process implies that the accuracy of the 3D printing is not a critical parameter. The plaster used here was provided by Omnicast: it is a special plaster employed in metal microcasting and is usually employed in goldsmithing and in metal casting more generally; this plaster is able to stand temperatures of up to 1000 °C.

**Figure 5:**
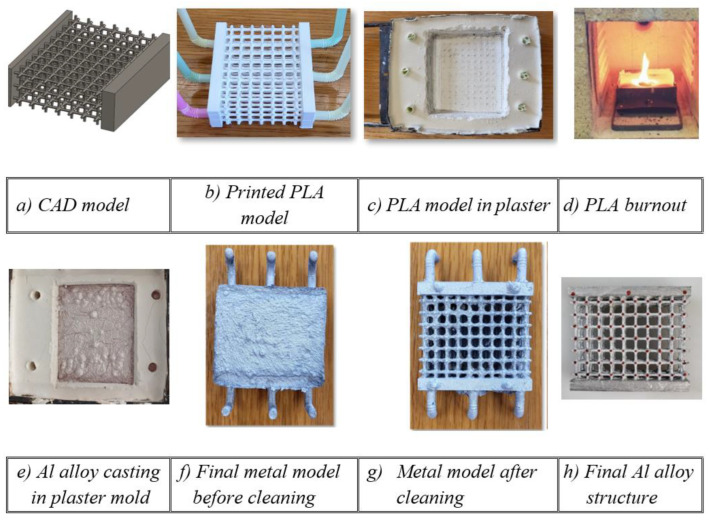
Pictures illustrating the various steps of the lattice creation process.

Finally, after the Al alloy casting and solidification, the process requires washing, cleaning and removal of excess metal in order to obtain the final structure. Further details can be found in [51]. A sequential outline of the manufacturing steps is given in Figure 5. The three types of produced samples and their geometrical characteristics are given in Figure 3.

**Figure 6:**
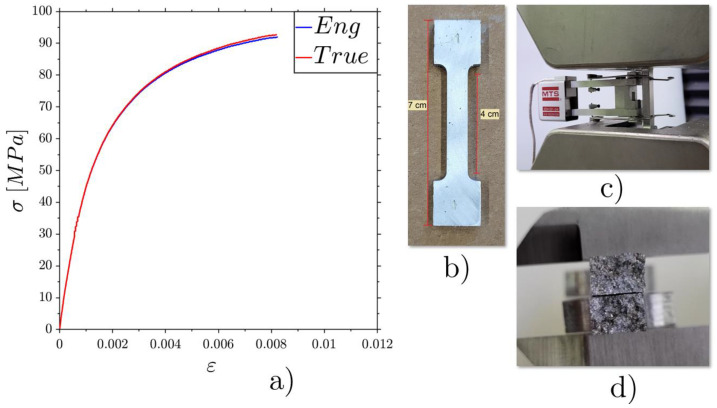
Engineering and true σ-ε curves for the tensile test (**a**), the dog-bone Al 6082 sample before the tensile test (**b**), the extensometer (**c**) and a cross-section view after fracture (**d**).

In order to compare the experimental results with the numerical ones, a tensile test on a dog-bone sample was carried out to characterize the base material (AA 6082 alloy) after the same manufacturing procedure. The tensile curve is reported in Figure 6a. Figure 6 also shows the sample before the tensile test (b), the experimental set-up (c) and a cross-section view of the fracture surfaces (d). From this figure, it is possible to observe the low ductility of the material, evident both from the low deformation obtained after the tensile test (Figure 6a) and from the kind of fracturing, with bright areas and necking absence.

The tensile results allowed us to obtain a stress–strain curve for the material for importation into the FE software. As is evident from the graph in Figure 6a, the elastic regime was relatively short, extending up to 30/35 MPa. The material subsequently started the plastic trend, maintaining a fairly constant increase in load until the fracture load (as high as 93 MPa) was reached. The resulting Young’s modulus was 57.8 GPa. This value is considered acceptable, lower than the theoretical modulus, which was about 70 GPa, due to the presence of porosity introduced by the casting process.

**Figure 7:**
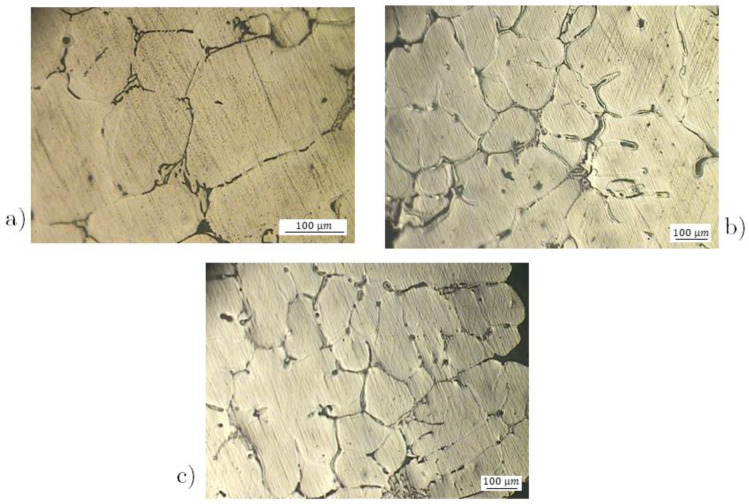
Microstructures of the Al 6082 alloy after the casting process: (**a**) 200X, (**b**) 100X and (**c**) 50X.

To characterize the microstructure, metallographic investigations were performed. Samples taken from the reticula structures were embedded in a classical bicomponent thermosetting resin to facilitate the polishing process with grinding paper and diamond paste. The polished sections were etched in Keller solution (0.5% HF and distilled water), and the microstructure was investigated with a metallographic Leitz optical microscope at 100× and 200× magnifications. The micrographs in Figure 7 show the microstructure of the sample at different magnifications: there are the interdendritic space of *α*-Al solid solution and the precipitates of the intermetallic phases on the edges of the interdendritic cells. This second phase formed during the casting and consists of Mg_2_Si, *β*-AlFeSi and *α*-AlFeMnSi. The metallographic structure is in good agreement with literature data [52]. In the micrographies of Figure 7 also appear the shrinkage cavities due to the casting process.

## 4. Experimental Tests and Finite Element Analysis

This section deals with the static and dynamic compression tests on the lattice structures previous defined in Figure 3.

**Figure 8:**
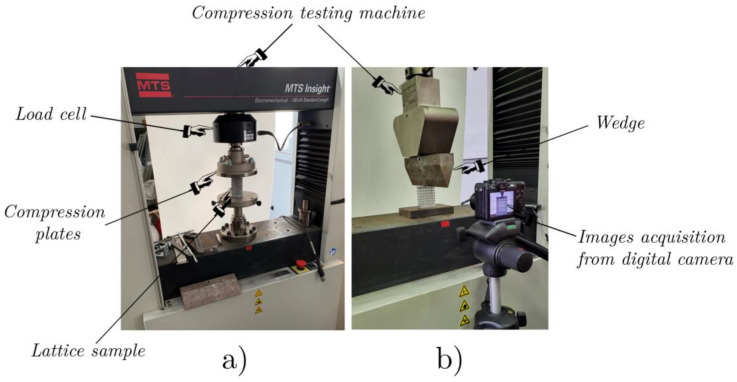
Experimental set-up for flat (**a**) and wedge (**b**) compression tests.

To investigate the mechanical response when varying the lattice density and to examine the role of the fillet radius on mechanical strength, both static flat (Figure 8a) and wedge (Figure 8b) compression tests were conducted.

The static flat compression test is a classical test performed on lattice structures to analyze their mechanical response in terms of their overall force–displacement behavior during progressive collapse. The tests were carried out in a compression machine with the adoption of a couple of compression plates (Figure 8a), keeping a constant crosshead speed of 1.25 mm/min during the elastic–plastic compression. The resulting forces were measured by means of the load cell shown in Figure 8a.

The static wedge compression test is not commonly used for lattice structures; the choice of this test was driven by the interest to examine the field of the stress and deformation distributions in the lattice structures when subjected to a concentrated load, which represents the most extreme scenario for this type of structure.

For each experimental test, an FE model that simulated it was generated to assess the reliability of the numerical model with respect to the prediction of the mechanical response and collapse. Experimental and numerical results were compared in terms of force–displacement responses and deformed configurations by means of Digital Image Correlation (DIC).

**Figure 9:**
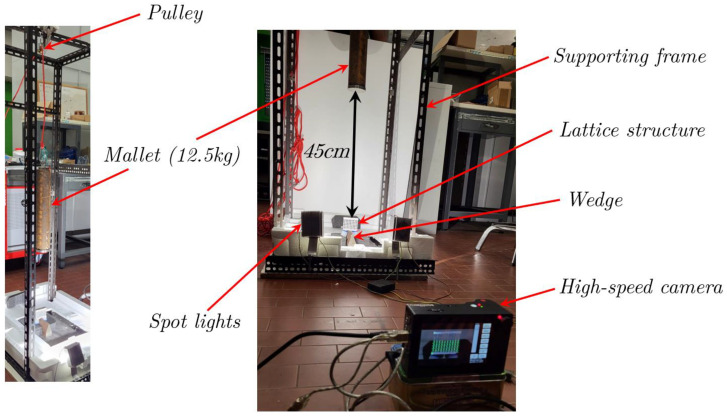
Experimental set-up for impact tests.

Dynamic compression tests are used to investigate mechanical responses under high strain rates. All impact tests were conducted with a drop-weight system, with a fall height of 450 mm (the mallet weight was 12.5 kg) and a resulting impact speed of about 3 m/sec; the deformation sequences were captured using a high-speed digital camera. The experimental set-up for the dynamic tests is shown in Figure 9.

### 4.1. Flat and Wedge Static Compression Tests

The first static compression test was performed on the low-density lattice structure (Figure 3a). For the flat compression tests, the upper flange was 2 mm thick for all the specimens.

The test was conducted under displacement control, with a very low velocity of the crossbar equal to 0.6 mm/min given the slenderness of the structure.

**Figure 10:**
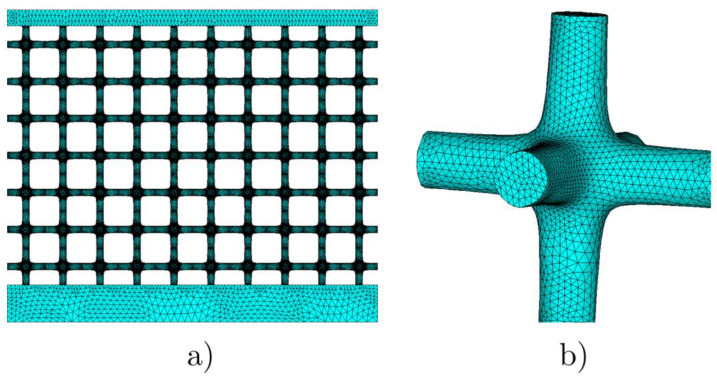
FE mesh of the low-density lattice (**a**) and detailed mesh of the unit cell (**b**).

The experimental tests were compared with the results obtained by finite element analysis and performed using solid 10-node tetrahedral elements. The mesh used for the lattice is shown in Figure 10, involving over four million elements. The FE analyses were performed, taking into account large displacements and material nonlinearity modeled as isotropic hardening von Mises plasticity. The experimental material response of Figure 6 is employed within the modelling.In all the FE models, the bottom flange was fully constrained.

The FE model for the flat compression tests shown in Figure 10a considered progressive vertical displacements of the top flange. The numerical model of the wedge compression test involved vertical displacements applied at the wedge, which was initially in contact with the upper flange; the surface-to-surface frictionless contact was modeled through contact-gap elements with an admissible interpenetration of 0.1 mm.

The experimental and FE results were compared on two fronts, the overall force–displacement responses and the internal displacements. The latter were measured in the experiments using the Digital Image Correlation (DIC) technique. To perform the DIC, the data acquisition rate was set at 10 images every 14 s, for a total of 85 images, which was largely sufficient to reliably follow large deformations.

**Figure 11:**
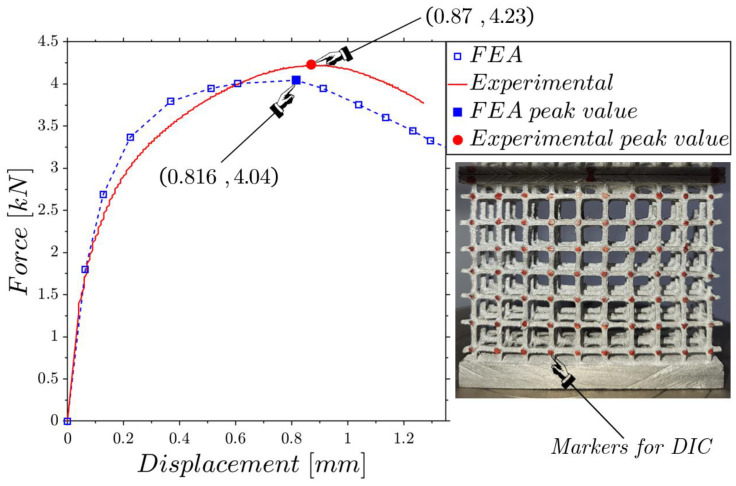
Comparison of the force–displacement response between the experimental test and the FEA for the low-density lattice (Figure 3a).

Figure 11 shows the experimental and FE force–displacement responses of the low-density lattice. The first branches of both curves almost overlap and then move away while maintaining a coherent trend. The peak values are very close, presenting a relative difference of 6.21% for displacement and 4.49% for force, showing the very close agreement between the experimental test and the numerical modeling.

**Figure 12:**
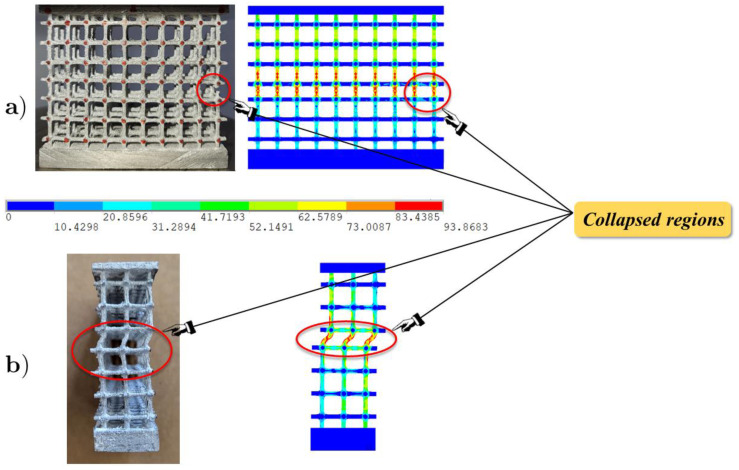
Front (**a**) and lateral (**b**) views of the experimental and FE deformed configurations at the incipient collapse.

Figure 12 shows the experimental and FE front and lateral views of the configuration corresponding to the peak value, highlighting the capability to identify the collapsed regions, which are concentrated in the most stressed central area.

**Figure 13:**
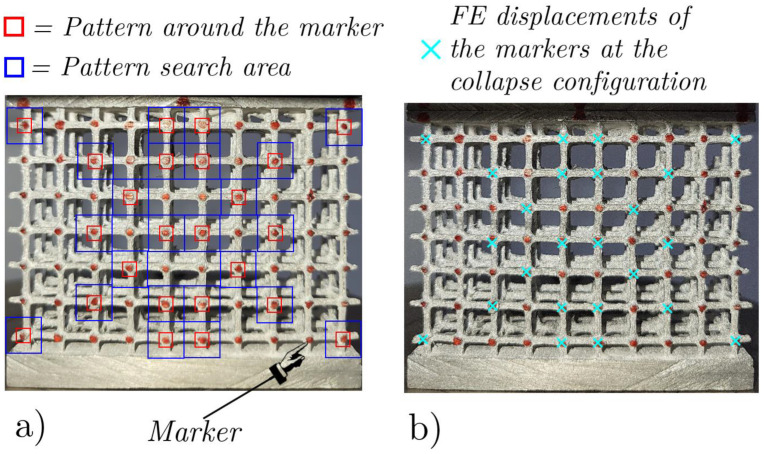
Marker patterns for DIC processing of the undeformed configuration of the low-density lattice (**a**) and the deformed configuration of the incipient-collapse configuration and FE displacements (cyan crosses) of the marker points (**b**).

A comparison of the internal displacements among the experiment and FEA is shown in Figure 13, where the DIC is used to follow the displacements of the marked points of the lattice during the loading. In Figure 13a, the red squares denote the designated patterns to be looked for within the blue rectangles in all the pictures in the sequence (85 for the case shown in Figure 13), employing the algorithm described in [53], which leverages cross-correlation. A major difference between the experimental results and the numerical predictions of displacements can be observed along the fourth row of Figure 13b; this deviation arose from some unpredictable internal defects of the specimen which significantly influenced the deformation behavior of the lattice structure. Such factors include internal material defects like microcavities or impurities stemming from the casting process, which make the material’s behavior more brittle than previewed. Conversely, numerical analyses operate with an idealized model, homogeneous at all points—an assumption that does not reflect real complexity. Furthermore, the FE model did not take into account the breakage of the elements that actually occurred during the experiments. However, our aim is to investigate the reliability of the ideal FE model in representing the phenomena under investigation, without taking into account the difficult predictability of sample defects that are generated during their production.

To quantify this discrepancy, we used the root mean square error (RMSE):

(7) $$RMSE=\sqrt{\frac{1}{N}\;\overset{N}{\underset{i=1}{\sum }}{\left(X_{i}^{exp}-X_{i}^{FEA}\right)}^{2}+{\left(Y_{i}^{exp}-Y_{i}^{FEA}\right)}^{2}}$$

where $X_{i}^{exp}\;\mathrm{and}\;Y_{i}^{exp}$ are the experimental marker coordinates and $X_{i}^{FEA}\;\mathrm{and}\;Y_{i}^{FEA}$ are the respective coordinates obtained from the FEA.

**Figure 14:**
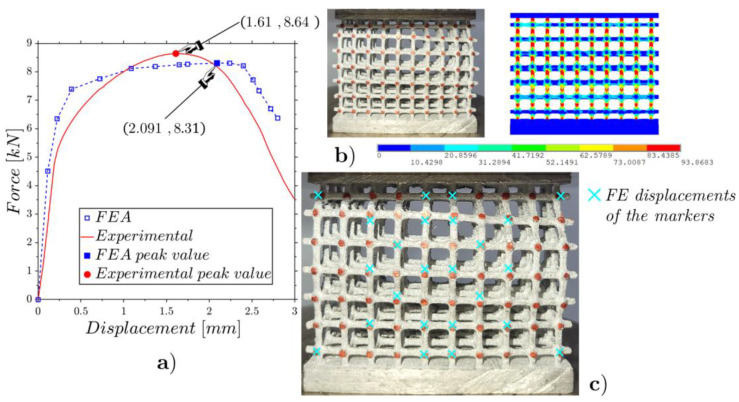
Flat static compression of the mean-density lattice (Figure 3b): experimental and FE force–displacement responses (**a**); experimental and FE configurations at the incipient collapse (**b**); comparison of the experimental and FE marker displacements (**c**).

**Figure 15:**
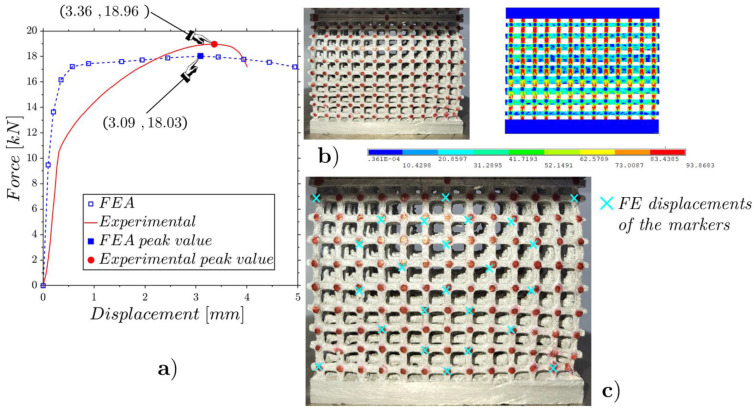
Flat static compression of the high-density lattice (Figure 3c): experimental and FE force–displacement responses (**a**); experimental and FE configurations at the incipient collapse (**b**); comparison of the experimental and FE marker displacements (**c**).

The RMSE of Figure 13b is computed on 24 markers (Figure 13a), the result being around 0.1 mm, which is about 11% of the displacement at the peak force shown in Figure 11. The results of the flat compression tests on the mean- (Figure 3b) and high-density lattices (Figure 3c) are shown in Figure 14 and Figure 15. For both tests, the experimental and FEA peak values are very close.

It is evident in Figure 14b and Figure 15b that curvatures occur in the images at the boundaries of the specimens. These curvatures, stemming from a perspective view, may potentially affect DIC analyses. Consequently, we selected the points where such visual distortions are absent. The RMSEs of the results for the internal displacements corresponding to Figure 14c and Figure 15c were 0.55 mm and 0.87 mm, which correspond to 26.30% of the peak displacements for the mean-density lattice and 28.16% of the peak displacements for the high-density lattice. These percentual results are just over double that obtained previously for the low-density lattice. This was due to the fracturing of some rods that occurred during the tests, which was not foreseen in the FE model.

From Figure 11, Figure 14 and Figure 15, it is evident that in all three cases, the peak force values obtained from the numerical simulations were slightly lower compared to the measured ones. The case that showed greater fidelity between the ideal numerical model and the tests was the low-density structure. This could be attributed to the fact that with a lower density there is a lower probability of defects being present. In assessing the peak load prior to collapse, it was encouraging to note that the values predicted by the numerical model slightly deviated from the experimental data. This suggests that, despite differences between experimental data and numerical simulations, the test results are comparable, and numerical models provide valuable insights on the design phase, and the capability to compare different lattice configurations.

**Figure 16:**
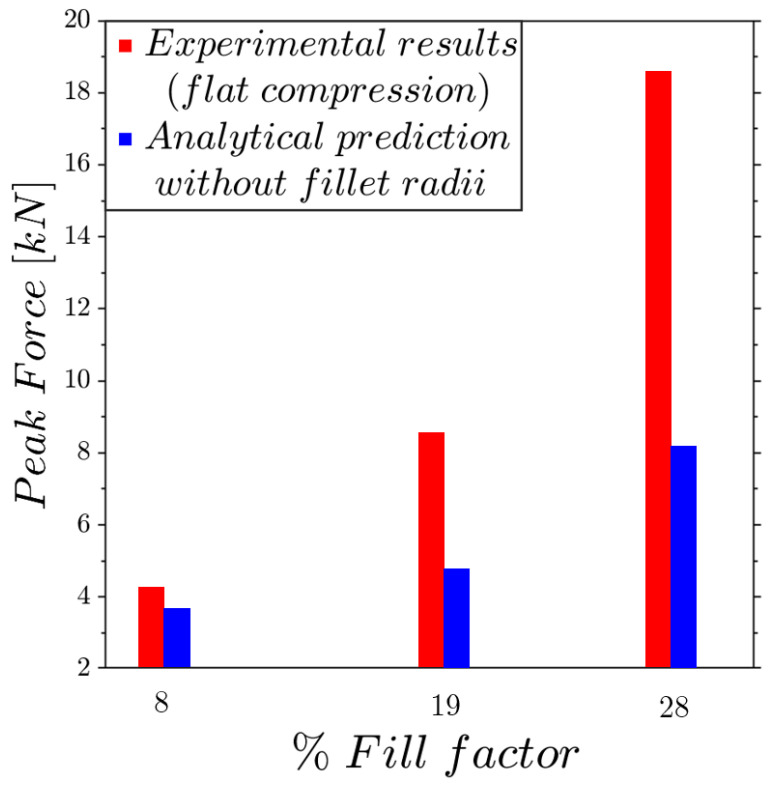
Experimental and analytical prediction of the dimensionless peak force trend under flat compression as a function of the fill factor.

Previous tests have demonstrated the high structural strength exhibited by these structures. Therefore, it was fundamental to investigate the effect of the fillet shape on the mechanical strength. A simple and effective approach to achieve this was by an analytical estimation of the maximum theoretical force that a structure could support before collapse. Consider the lattice without the presence of the fillet radii; if it is cut by a horizontal plane, the vertical rods will support the load; hence, the maximum force is simply determined by multiplying the tensile ultimate strength of 93 MPa by the sum of the areas of the cut rods. In Figure 16, the peak forces measured by the experiments are compared with the theoretical peak values. The results are interesting and show that the experimental trend tends to improve as the filling increases more than theoretically expected.

This more favorable trend can be explained by the fact that the analytical calculation relies on a purely uniaxial stress state, whereas in the actual lattice structures, the fillet shape, which forms the main part of the unit cell, results in a triaxial stress state, leading to a significant enhancement of mechanical strength.

The experimental tests were repeated for a wedge compression, which is not commonly applied to lattice structures. However, we assert its significance, as it offers insight into the propagation of plastic flow under concentrated loads and elucidates the early onset of collapse compared to scenarios with a uniform load distribution.

For the wedge compression tests, the upper flange was taken to be 5 mm thick for all the lattices in order to avoid premature failure of the flange before the collapse of the lattice occurred.

**Figure 17:**
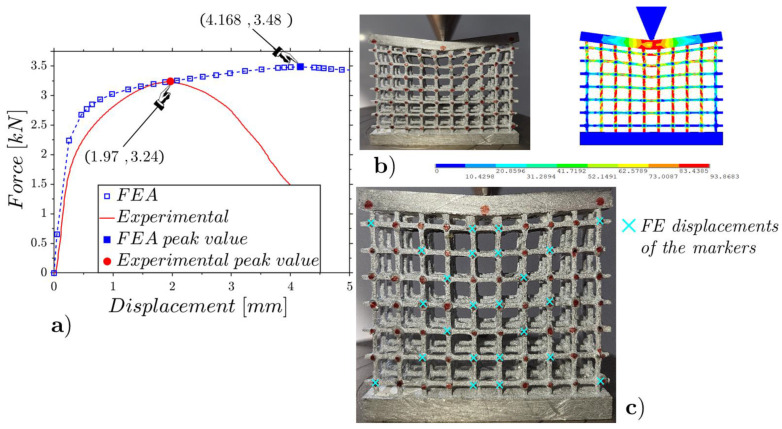
Wedge static compression of the low-density lattice (Figure 3c): experimental and FE force–displacement responses (**a**); experimental and FE configurations at the incipient collapse (**b**); comparison of the experimental and FE marker displacements (**c**).

**Figure 18:**
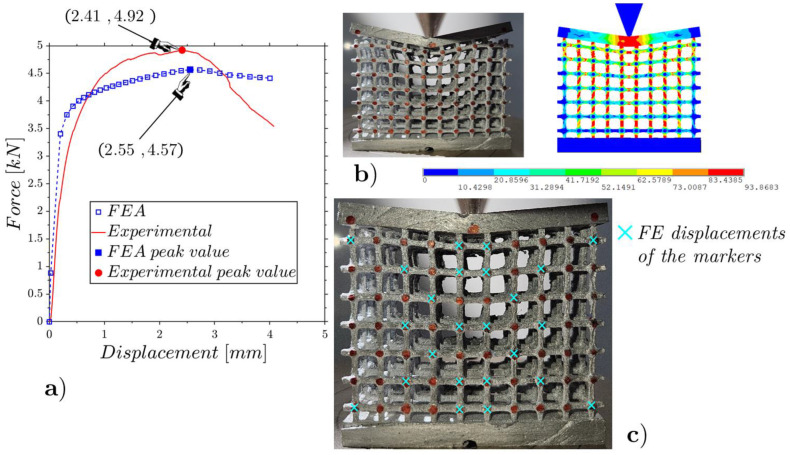
Wedge static compression of the mean-density lattice (Figure 3c): experimental and FE force–displacement responses (**a**); experimental and FE configurations at the incipient collapse (**b**); comparison of the experimental and FE marker displacements (**c**).

**Figure 19:**
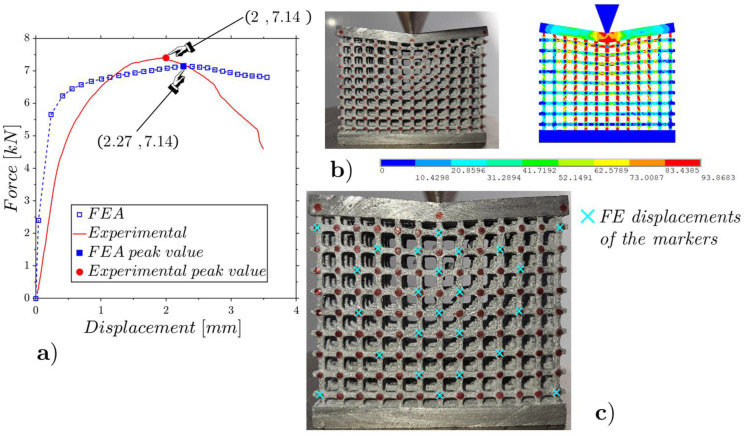
Wedge static compression of the high-density lattice (Figure 3c): experimental and FE force–displacement responses (**a**); experimental and FE configurations at the incipient collapse (**b**); comparison of the experimental and FE marker displacements (**c**).

The results are shown in Figure 17, Figure 18 and Figure 19 for the low-, mean- and high-density specimens, respectively. The comparisons between the experimental results and the FEAs regarding the deformed configurations and internal displacements refer to the configurations at the incipient collapse.

The low-density lattice in Figure 17 shows a notable discrepancy with the results predicted by the numerical model, highlighting that the low-density lattice is not suitable to support high concentrated loads. The other two specimens with mean and high densities, which evidence trends close to those expected from the numerical models, showing to be capable of supporting high concentrated loads. 

The ratios among the RMSEs of the relative internal displacements for the experiments and FEAs (Figure 17c, Figure 18c and Figure 19c) and the relative displacements for the peak force results of 12%, 10% and 9% are smaller than the cases under flat compression due to the lower deformations that emerged in the wedge compression tests.

**Figure 20:**
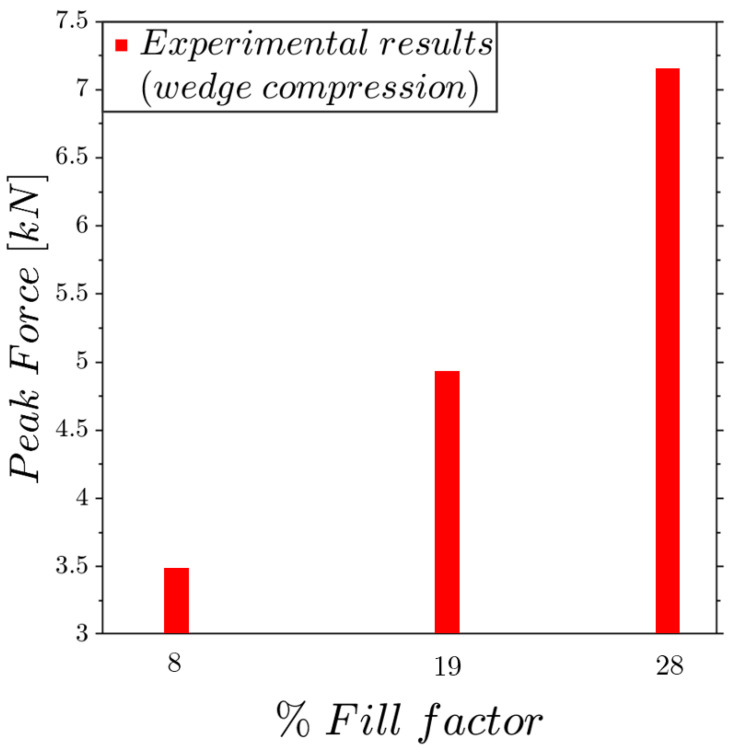
Experimental dimensionless peak force trends under wedge compression as a function of the fill factor.

For the wedge compression tests, it is interesting to evaluate the dimensionless ratios between peak forces and lattice weights varying as a function of the fill factor, as re-ported in Figure 20. Unfortunately, in this load case, there is not a simple analytical solution for the comparison as for the flat compressions. Figure 20 shows that for wedge compression loads, the increase in the fill factor does not improve the maximum strength, highlighting how this lattice structures suffer under the application of high concentrated loads.

### 4.2. Flat and Wedge Dynamic Compression Tests

In this section, the flat and wedge impact compression tests are discussed.

The experimental set-up, shown in Figure 9, involved the specimen being positioned in equilibrium on the wedge (or the flat supporting plane) whose center was collinear with the direction of the 12.5 kg mallet. The duration of the dynamic event was very short, less than 50 ms, which did not allow us to perform accurate DIC examinations. The evolution of the impact was captured by a high-speed camera using 5000 frames per second.

**Figure 21:**
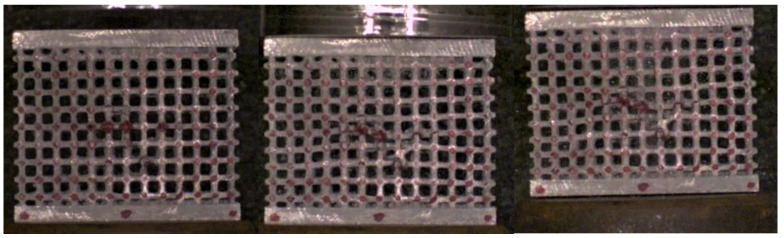
Sequence frames of the flat compression impact test on the high-density specimen.

The first test (Figure 21) was a classic, well-known one, which involved a flat impact compression of the specimen with a high density; the structure withstood the impact, responding with a uniform deformation throughout its entire extension, undergoing a small fragmentation involving only the central part and hence demonstrating its ability to effectively absorb the impact energy.

**Figure 22:**
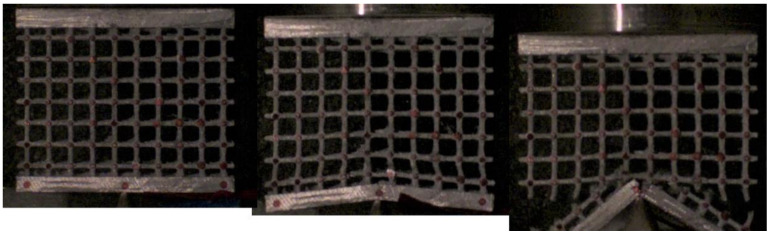
Sequence frames of the wedge compression impact test on the low-density specimen.

**Figure 23:**
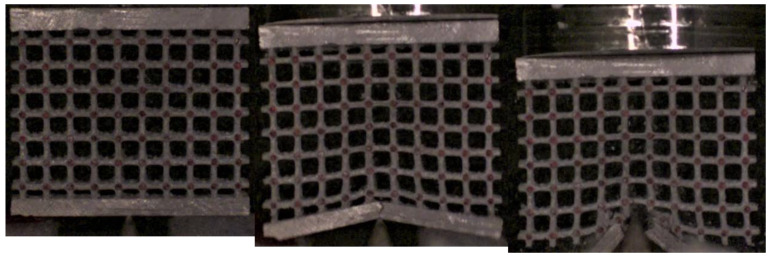
Sequence frames of the wedge compression impact test on the mean-density specimen.

**Figure 24:**
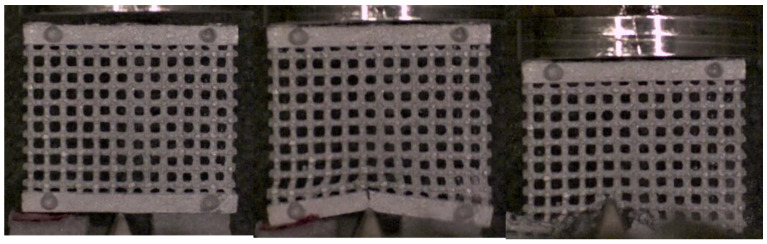
Sequence frames of the wedge compression impact test on the high-density specimen.

It was more interesting to investigate what happened when the impact involved a high concentrated load. Figure 22, Figure 23 and Figure 24 show the sequence frames for the wedge compression impact tests on the low-, mean- and high-density specimens, respectively. In all three cases, at the first impact stage, the central lattice regions deformed and absorbed the kinetic energy of the falling mallet; in the subsequent phases, the structures collapsed, leading to progressive and unstable fragmentation involving the entire lattices.

In other words, the tests clearly revealed a significant change in the structures’ behavior, bringing out an important sensitivity to load extension and speed (i.e., strain rates). In the static tests, the structures subjected to flat and wedge compressions responded primarily within the plastic field. Conversely, under impact tests, an initial, small plastic deformation was observed, but, especially when the impact occurred with the wedge, it was followed by sudden fragmentation, indicating a transition to markedly brittle behavior.

## 5. Conclusions

This work aims to explore how to improve the mechanical strength-to-weight ratios of metal cubic lattice structures by means of a fillet shape for the unit cells inspired by triply periodic minimal surfaces. The analyzed lattice structures were realized in aluminum by means of the lost-PLA processing technique, a method similar to the well-known “lost wax” technique, in which PLA is substituted for the wax.

The core of the study is experimental, and the flat and wedge compression tests were performed statically and dynamically on three different samples, varying the fillet shape and the fill factor. For each static test, a numerical simulation was carried out by means of finite element analysis in order to understand how the numerical prediction of an ideal (defect-free) specimen could be consistent with the experiments. The tests and finite element analyses were compared on two fronts: the force–displacement responses and the deformed configurations, evaluated from experiments using Digital Image Correlation to capture the displacements of the markers on the lattices.

The peak force was very well predicted by the numerical simulations. However, the agreement reduced for the punctual displacement evaluations and the overall force–displacement curves due to the multiple defects present in the real samples. The discrepancies between the experimental and numerical results were attributed to unpredictable factors, such as internal material defects and impurities, that caused a premature collapse of the lattices, while the numerical analyses assumed homogeneity and overlooked element fractures observed in the experiments.

The benefit of the triply periodic minimal surface-type fillet shape is evaluated through simple analytical considerations for the case of flat compression, involving a lattice structure without fillet radii. The results, in terms of the mechanical strength as a function of the fill factor, evidence a great benefit of the proposed fillet shape, which increases when the fill factor grows more than analytically expected given the results for lattices without any fillet radii. The discrepancy arises because analytical calculations assume a uniaxial stress state, whereas in lattice structures, the fillet shape, which is the primary feature of the unit cell, induces a triaxial stress state that significantly enhances the mechanical strength.

The dynamic tests show a great resistance of lattices to flat impact. When wedge impact is accounted, which involves high concentrated-loads, the lattice structure exhibits a notable sensitivity to strain rates; it shows an initial minor plastic deformation, swiftly succeeded by abrupt fragmentation, evidencing a switch towards brittle behavior.

The agreement between experimental and FEA results in terms of mechanical strength indicates that the search for optimal shapes can partially be conducted through numerical analysis, particularly with regard to the optimal choice of the parameters, a and b, defining the TPMS fillet shape.

## Data Availability

Data are contained within the article.
